# From Spurious Interference to Biological Signal: Repurposing Weather Radars to Monitor and Study the Amazonian Avifauna

**DOI:** 10.1002/ece3.71860

**Published:** 2025-07-29

**Authors:** Maria C. T. D. Belotti, Victória de Souza Wojahn, Carlos Augusto Morales Rodriguez, Kyle G. Horton

**Affiliations:** ^1^ Department of Fish, Wildlife, and Conservation Biology Colorado State University Fort Collins Colorado USA; ^2^ Department of Civil and Environmental Engineering Colorado State University Fort Collins Colorado USA; ^3^ Institute of Astronomy, Geophysics, and Atmospheric Sciences University of São Paulo São Paulo SP Brazil

**Keywords:** Amazon Rainforest, bird migration, communal roosting, Hirundinidae, phenology, radar ornithology

## Abstract

Many species of swallows and martins congregate in large nonbreeding aggregations throughout the Americas. These roosts typically occur for several days to weeks in the same place during the same time of the year and disappear suddenly as the birds continue their migratory journeys. In the Amazon Rainforest, however, there are reports of large communal roosts of varying species composition throughout the year. Due to the high biomass density of these aggregations, we can systematically observe these tropical roosts using data collected by the operational S‐band Doppler weather radar located in Manaus (3°08′56.0″ S, 59°59′29.1″ W) Using data collected by this radar over 2 years (2014, 2015), we describe the temporal and spatial patterns of roost size in the Amazon Rainforest, and compare it to a similar dataset collected in the Great Lakes region of North America, where swallows and martins form pre‐migratory roosts. Our findings confirm that roosting activity occurs throughout the year in the region around Manaus, and thus likely gather multiple species of swallows and martins. The peak of roosting activity in both years occurred from January to May, when observations on the ground suggest that roosts are predominantly Purple Martin aggregations. We found that the average daily number of birds in roosts in Manaus in 2015 is up to 7 times larger than what was observed in the Great Lakes, even though the area of the latter is 8.7 times larger than the area sampled around Manaus. Our findings highlight the significance of the Amazon Rainforest for swallow and martin populations. Because this region hosts migrating individuals from the Nearctic‐Neotropical and the Austral migratory systems, resident species may share these roosts with populations from both systems during separate times of the year, creating an indirect link between the two poles of the Americas.

## Graphical abstract

In our study, we processed and analyzed data collected from 2014 to 2015 by a radar located in Manaus, AM, in the Amazon Rainforest, comparing it to a similar dataset from the Great Lakes region in North America. Our results suggest that the region surrounding this radar hosted up to seven times more swallows and martins in communal roosts than the entire Great Lakes region, which is 8.7 times larger. We describe roost locations and phenology, discussing how these large roosts are connecting migrant populations from North and South America.

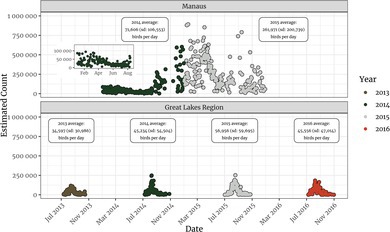

## 1 Introduction

Every year, billions of migratory birds travel thousands of kilometers between their breeding and nonbreeding grounds. The fact that these two phases of these species' annual cycle occur in different places means that their population sizes could be influenced by conditions they encounter during migration or in entirely different regions of the world (Newton 2004; Sherry and Holmes 1996). Moreover, conditions experienced in one season could affect survival and fecundity in the following season, resulting in complex seasonal interactions that ultimately have important population ramifications (Harrison et al. 2011). Additionally, since individuals that breed together could spend their nonbreeding period in very distant regions, observing the effects of these seasonal interactions can be challenging (Webster et al. 2002; Cristol et al. 1999). These complexities create obstacles for researching and conserving migratory species, especially when basic knowledge about what they do during their time in the tropics is lacking. Within the Americas, for example, knowledge about the nonbreeding geographic range limits and habitat associations of some Nearctic–Neotropical migrants such as the Connecticut Warbler ( *Oporornis agilis* , refer to Table S1 for a dictionary of species names), or the Chimney Swift (*Cheatura pelagica*) is incomplete or based on very few records (Wolfe et al. 2012; Gomes 2023; Steeves et al. 2020). This lack of knowledge is even more dramatic in the case of Austral migrants, for which data are scarce throughout the annual cycle (Faaborg et al. 2010a; Chesser 1994; Jahn et al. 2004).

The literature suggests many reasons for these knowledge gaps, such as challenges associated with asymmetric collaborations between north and south scientific institutions (Malhado et al. 2014), a tight link between national identity and science funding, particularly during the Cold War (Wagner et al. 2015), a perception amongst ornithologists that the tropical nonbreeding period of migrants was less important (Faaborg et al. 2010b), and the lack of an integrative conceptual framework that would allow us to develop new hypotheses and research questions about the full annual cycle of migratory species (Marra et al. 2015; Bowlin et al. 2010; Faaborg et al. 2010a). These cultural and structural challenges are being overcome (Wagner et al. 2015; Malhado et al. 2014; Perez and Hogan 2018); however, one barrier remains unaltered: one of the most important nonbreeding regions of the Americas, the Amazon Rainforest, is mostly inaccessible for systematic long‐term field studies which would be needed if we were to fill some of these gaps (Lees et al. 2020).

Swallows and martins epitomize many of these problems. Species that reside in North America during their breeding period, such as Tree Swallows ( *Tachycineta bicolor* ), Purple Martins ( *Progne subis* ), and Barn Swallows ( *Hirundo rustica* ), are relatively well‐studied and, like other species of aerial insectivores, have been facing important breeding population declines (Rosenberg et al. 2019; Tautin et al. 2017). However, knowledge about the period they spend in the Neotropics is still deficient, particularly for species that spend their nonbreeding period in the Amazon Rainforest such as the Purple Martin. Additionally, since many of the species of the *Progne* genus are virtually indistinguishable in the field, new locations are still being added every couple of years to the nonbreeding ranges of the intertropical migrants of this genus, such as the Caribbean Martin ( *Progne dominicensis* ) and the Cuban Martin ( *Progne cryptoleuca* , García‐Lau et al. 2021; Perlut et al. 2017; Cooper 2024). At last, Austral migrants that breed in southern South America and spend their nonbreeding period in the Amazon Rainforest, like the Southern Martin ( *Progne elegans* ) and the Brown‐chested Martin subspecies fusca ( *Progne tapera fusca* ), have basic life history, phenology, and distribution knowledge gaps. Community science data (Santos et al. 2021), stable isotopes (Imlay et al. 2018), and light level geolocators (Fraser et al. 2012) have allowed us to advance towards closing some of these gaps, but data are still insufficient to understand, for example, how these species are responding to recent extreme droughts and widespread wildfires in the Amazon, and what can be done to reverse the declining trends in breeding populations in the Northern Hemisphere.

Weather radars can circumvent some of the challenges associated with the remoteness of the Amazon Rainforest, allowing us to collect data on the location, timing, altitude, and individual density of mass aerial movements for a broad range of taxa. In Europe and the United States, for example, data collected by operational weather radars have been used to observe, monitor, and predict nocturnal bird migration at a continental scale (Bauer et al. 2018, see for a comprehensive review), allowing us to reframe our conceptual understanding of these seasonal movements (Heffernan et al. 2014; Kelly and Horton 2016). Additionally, radars have also been used to study emergences of aerial insects, which are notoriously difficult to observe (Bauer et al. 2024; Tielens et al. 2021). In these examples, radars can offer us information about biological events, but they lack the taxonomic precision of on‐the‐ground studies. However, in some unique instances, when use of the airspace at a given time and space is taxon‐specific, we can refer to ancillary life history knowledge to infer the family or species observed by the radars. This is the case, for example, of Mexican free‐tailed bats ( *Tadarida brasiliensis* ), whose enormous dispersals from well‐known caves and bridges in Texas are systematically captured by local radars (Frick et al. 2012; Horn and Kunz 2008; Stepanian and Wainwright 2018).

In addition to select bat movements, nearby weather radars systematically capture swallows and martins due to their distinctive communal roosting behavior. When they are not breeding, swallows and martins congregate at night in large aggregations that can gather hundreds of thousands of birds. These aggregations can have one or multiple species of Hirundinidae, and their early morning dispersal creates a unique doughnut‐shaped high reflectivity signature on radar reflectivity renderings, which can be used to quantify roost size, location, date, and timing (Belotti et al. 2023; Kevin Richard Russell 1996). In their temperate breeding grounds in North America, swallows and martins (most notably, but not exclusively, Purple Martins and Tree Swallows) join roosts as a pre‐migratory staging strategy as they refuel and prepare for their southbound migration (Warnock 2010; Belotti et al. 2023; Bridge et al. 2015; Russell 1996). With data from the NEXRAD network of weather radars from the United States, researchers have been able to extensively map and study these roosts and systematically measure the ability of weather radars to detect them (Russell et al. 1998; Bridge et al. 2015; Kelly and Pletschet 2017). In particular, studies conducted in the North American Great Lakes have quantified roost size and persistence in the landscape, whereas also demonstrating that the roosting season has been advancing in the past 21 years, possibly due to climate change (Belotti et al. 2023; Kelly et al. 2012; Deng et al. 2023).

These communal roosts are not exclusive to North America: throughout South America, but particularly in the Amazon Rainforest, communal roosts gather most of the swallow and martin species mentioned up until now, except for Tree Swallows and possibly Caribbean Martins and Cuban Martins. Between November and late May, roosts in the Amazon gather Purple Martins during their nonbreeding period, and resident Brown‐chested Martins subspecies *tapera*, with occasional reports of smaller numbers of Barn Swallows and Gray‐breasted Martins (Sick 2001; Arruda 2023; Brown et al. 2021; Turner 2025, *Progne chalybea* ). From July until September, when the Austral migrants establish themselves in the Amazon for their nonbreeding period, roosts of Southern Martins and Brown‐chested Martins subspecies *fusca* begin to occur (Oren 1980; Turner 2020, 2025). These aggregations in the Amazon Rainforest, contrary to their North American counterpart, gather species during their nonbreeding period when birds are under different ecological and behavioral constraints. Examining these differences in comparative studies could help us better define these constraints and determine the full migration chronologies of these species when they all but disappear into the vastness of the Amazon.

To fill this gap, we give the first description of swallow and martin roosts in Brazil based on data collected by a radar in Manaus, in the state of Amazonas, Brazil, from 2014 to 2015. These 2 years of data are used to describe the yearly phenology of swallow and martin roosting behavior in the Amazon Rainforest. We also describe the spatial distribution of these aggregations, and how they vary within and across years. Additionally, we will compare daily activity in the nonbreeding roosts observed in the region around Manaus with that of pre‐migratory staging roosts gathering in the North American Great Lakes from 2013 to 2016 (accounting for two full annual cycles). Because the Amazon Rainforest hosts swallows and martins migrating both from North America and from southern South America, we hypothesize that, contrary to the dynamics observed within the Great Lakes, roosting activity in Manaus will occur throughout the year. At last, since a well‐documented roost composed predominantly of Purple Martins (but also gathering significant numbers of Brown‐chested Martins) occurs close to Manaus, in the Negro River, from January to May, we expect to observe a peak in roosting activity during that time (Arruda 2023; Oliveira 2021).

## 2 Materials and Methods

### 2.1 Study Region

Wetlands correspond to approximately 34% of the total area of the Amazon basin (Wittmann et al. 2022; Junk et al. 2011). The region surrounding the urban area of Manaus is a mosaic of terra firme moist forests (forests that are not seasonally waterlogged), secondary forests, sparse pasture areas associated with roads, and two major rivers and their affluents, the Negro river and the Amazon/Solimões river, with their associated floodplains (Junk 1997; Olson et al. 2001, so called river‐floodplain systems). These river‐floodplain systems can be further divided into white water systems with high productivity due to high loads of suspended sediment and nutrients, such as the Amazon/Solimões river, and nutrient‐poor black water systems such as the Negro river (Junk et al. 2012, 2015). These biogeochemical differences result in marked heterogeneity of fauna and flora community composition between the floodplains of white water rivers, so‐called várzeas (singular: várzea), and floodplains of black water rivers, igapós (Junk et al. 2011, singular: igapó).

Floodplains in the Amazon basin are drained by a combination of overbank diffusive flow and channelized flows through numerous floodplain channels (Trigg et al. 2012). These floodplain channels play a key role in determining the hydrodynamic characteristics of river‐floodplain systems, which in turn regulate biological and biogeochemical processes in the Amazon basin. Due to its mostly flat landscape, wetlands in the Amazon lowlands are subject to a flood pulse of predictable magnitude and timing, with one high‐water and onelow‐water period during the year (Junk et al. 1989; Wittmann et al. 2022, see Figure S1 for plots characterizing this behavior). This monomodal flood pulse characterizes the flow regime in most of the Amazonian lowlands, that is primarily driven by precipitation in the upper catchment area, which explains the time lag in flood peaks that exists between upstream and downstream reaches (Correa et al. 2022). The flow of water from the main river to its network of floodplain channels creates a complex pattern of erosion and sedimentation that is driven by the differences between white and black water rivers and results in spatial heterogeneity in vegetation community composition. This heterogeneity, paired with the yearly disturbance of the flood pulse, gives rise to diverse seasonally available habitats and influences vertebrate species distributions and landscape connectivity within the alluvial landscape (Salo et al. 1986; Peterman 1997; Wittmann et al. 2022; Junk et al. 1989).

### 2.2 Data Extraction and Processing

For this study, we used a set of 2 years of volumetric data collected by the S‐band Doppler weather radar located in the city of Manaus, Amazonas and made available to us by the Amazon Protection System (SIPAM, Sistema de Proteção da Amazônia). The technical specifications of the radar are summarized in Table 1. We downloaded and rendered all scans made between January 1st, 2014, and December 31st, 2015 using pyArt version 1.18.1 (Helmus and Collis 2016) for the Python Programming Language (version 3.11, maintained by the Python Software Foundation). We narrowed our search to an hour before and 2 h after local sunrise (approximately 5:00–8:00 in local time) which is usually when roost departures occur (Russell 1996; Belotti et al. 2023; Kelly and Pletschet 2017). We then used Label Studio to manually annotate each scan, drawing bounding boxes around each roost (Tkachenko et al. 2020). We also classified each dispersal as either clear roosts, roosts contaminated by precipitation or other sources of radar noise, or roosts that got mixed within a ring of mid‐level reflectivity around the radar called “ground clutter region”, in which the radar beams are still low and often capture trees and buildings. We also made notes for each day about the presence of precipitation or anomalous propagation that could have affected our ability to detect roosts if they were present (for an example of a roost caught in clutter and its vertical cross section, see Figure S2).

**TABLE 1:** Technical specifications of the S‐band Doppler weather radar located in Manaus, Amazonas.

Instrument parameter	Value
Location	3°08′56.0″ S, 59° 59′29.1″ W
Wavelength	0.10 m
Frequency	2.997925 × 10^9^ Hz
Horizontal beamwidth	1.8°
Vertical beamwidth	1.8°
Range gate	500 m
Volume coverage pattern	Fixed
Elevation angles	0.9°, 1.5°, 2°, 4°, 5°, 6°, 7°, 8°, 9°, 10.5°, 12°, 13.5°, 15°, 16.5°, 18°, 19°
Pulse width	105 μs
Pulse repetition time	0.00161 s
Unambiguous range	240,000 m
Nyquist velocity	15.525 m s^−1^
Azimuthal strategy	294 or 360 azimuths per sweep

After manual annotation, we used the coordinates of the bounding boxes to extract and filter reflectivity measurements within each roost. Because one of our roosts was frequently within the radar's ground clutter region, we designed clutter masks to remove some of the mid‐level reflectivity measurements surrounding the radar, which could artificially increase our roost size estimates. We did so by selecting scans within the lower 2% quantile of summed reflectivity within our study period. We used these scans to calculate two average masks of ground clutter reflectivity (in $cm^{6}\;km^{-3}$) for every elevation angle, for each azimuthal configuration (294 or 360 azimuths). We limited our masks to a region of up to 27.5 km from the radar, where we usually observed ground clutter during our manual screening. We then subtracted this mask from reflectivity measurements in every scan before estimating the number of birds within each bounding box. We refer the reader to Figure S3, where we plot the number of birds considered to be clutter for each sweep within each detection. We also removed from our analysis pixels that had very high (greater than 30 dBZ) or very low (lower than 5 dBZ) reflectivity since those are unlikely to correspond to swallows and martins. At last, since most of the roosting activity occurred at close range to the Manaus radar (see Figure S3), we did not account for range biases that are typical of radar data.

We estimated the number of birds within each roost following the method described in Chilson et al. (2012): we converted reflectivity factor ($Z_{e}$, measured in dBZ) within each radar sampling volume to linear scale ($mm^{6}\;m^{-3}$), and then transformed it into reflectivity (η), which can be interpreted as the density of scatterers of a given radar cross section (RCS) in the atmosphere (units are $cm^{6}\;km^{-3}$). We can obtain the radar cross section of a typical Hirundinidae from its mass by using the relationship proposed in Horton et al. (2019): $\log\left(\mathrm{RCS}\right)=0.699\times \log\left(\text{mass}\right)$. Since Purple Martins are the largest Hirundinidae in the Americas, we used their mass of 51 g (Dunning 2008), which corresponds to an RCS of 15.2 $cm^{2}$, to obtain conservative estimates of the number of birds. In dividing η by this RCS, we are left with the density in units of number of birds per $km^{3}$. The last step, thus, is to obtain the volume sampled by the radar at each azimuth (antenna rotation angle) and range.

Calculating this volume presents a challenge because the beamwidth of the Manaus radar is 1.8°, whereas its azimuthal strategy consists of performing a rotation of 1° (360 azimuths) or 1.22° (294 azimuths) between each azimuth, for each sweep. Because the rotation angle of both strategies is smaller than the radar beamwidth, there is a region between each pair of consecutive azimuths that is sampled twice. We accounted for this double sampling by using Monte Carlo integration to estimate the volume of the intersection between two radar sampling volumes in each azimuthal strategy, assuming that the radar sampling volume has the shape of a conical frustum of beamwidth 1.8°. We could then use the estimated intersection volume to calculate the number of birds within the two intersections ($N_{\text{left}}$ and $N_{\text{right}}$) and the number of birds within the single‐sampled region ($N_{\text{core}}$). The number of birds caught within each intersection was divided between the two corresponding azimuths so that the total number of birds per azimuth and range was equal to $\frac{N_{\text{left}}}{2}+N_{\text{core}}+\frac{N_{\text{right}}}{2}$. In doing this for all the azimuth and range combinations within each bounding box and summing the results, we get estimates of the number of birds within the roost dispersal at the time of each sweep. We restricted our counts to the first sweep (at an elevation angle of 0.9°), since sweeps at higher elevation angles either overlap the first one (due to the high beamwidth) or overshoot the roost dispersal.

The bounding boxes represent a single scan or frame of the dispersal event. A full dispersal typically happens across multiple scans and thus has multiple detections. The groups of detections that represent a single dispersal event are called tracks. We grouped detections into tracks by using a heuristic procedure that matched each detection with the one in the previous scan with which it overlapped the most. If a given detection overlapped more than one detection in the previous scan, we paired it with the nearest one. The results of this procedure were manually inspected for accuracy. We could then further summarize counts per track by extracting the peak count within each track. We summed counts across roosts on each day to obtain estimates of daily relative abundance. Because roosts often occur in the same place day after day, some regions accumulate roost occurrences throughout a season. In order to understand the temporal patterns of use of each roosting region within a year and between our two study years, we used mean shift clustering to find regions that were consistently used by swallows and martins to roost throughout our study period (this approach was first employed in Belotti et al. 2023).

In our analyses, we considered a day to have been sampled if it had at least 6 scans (approximately 1/3 of the expected 15 scans within our daily sampling period), and those scans were not radar failures and had no precipitation or anomalous propagation. If a scan had precipitation or anomalous propagation, but a roost was visible, we flagged it as a presence, but did not use it for our quantitative analyses of roost size.

We compared daily relative abundances and roost size distribution from Manaus with those observed in the Great Lake region of North America. For this purpose, we used a dataset from the same period collected from 12 operational S‐band radar stations in the Great Lakes region of the United States (Belotti et al. 2024). This dataset was created by running a machine learning pipeline that is capable of detecting roost signatures and tracking them as they disperse in the airspace. The output of this pipeline is bounding boxes, which were labeled following a protocol similar to what we just described for the dataset from Manaus. Reflectivity measurements were extracted from each bounding box using the same approach, except no clutter filtering was implemented (roosts were not typically found within the clutter region of the radars). To assert that the two datasets were comparable, we extracted the minimum and maximum detected reflectivity for each range gate for all scans made within our study period in the Manaus dataset, and for a sample of 10% of the scans from the Great Lakes region. We averaged measurements across scans to obtain one estimate of the minimum and one of the maximum detectable signals for each range gate (see Figures S4 and S5). We found that the NEXRAD radars were capable of detecting much lower reflectivity measurements than the radar from Manaus, indicating that the latter is more likely to underestimate the number of birds since it is not as sensitive to lower reflectivities.

## 3 Results

We found 308 days in 2014 and 205 days in 2015 with at least six scans available in the archive during our sampling window. In 2014, 12 days in November had to be removed due to radar failure. Precipitation or anomalous propagation led us to discard 118 days in 2014 and 160 days in 2015 in our estimation of roost size, even though roosts were observed on 78 of the contaminated days in 2014 and on 131 of the contaminated days in 2015. After removing days in which sampling did not satisfy our criteria due to radar failure or lack of scans, we were left with 178 sampled days in 2014 and 145 sampled days in 2015 for the purpose of estimating roost size. In our analysis of presence/absence of roosts, which combines clear and contaminated scans, we were left with 256 days (178 clear, 78 weather contaminated) in 2014 and 276 days (145 clear, 131 weather contaminated) in 2015.

**FIGURE 1:**
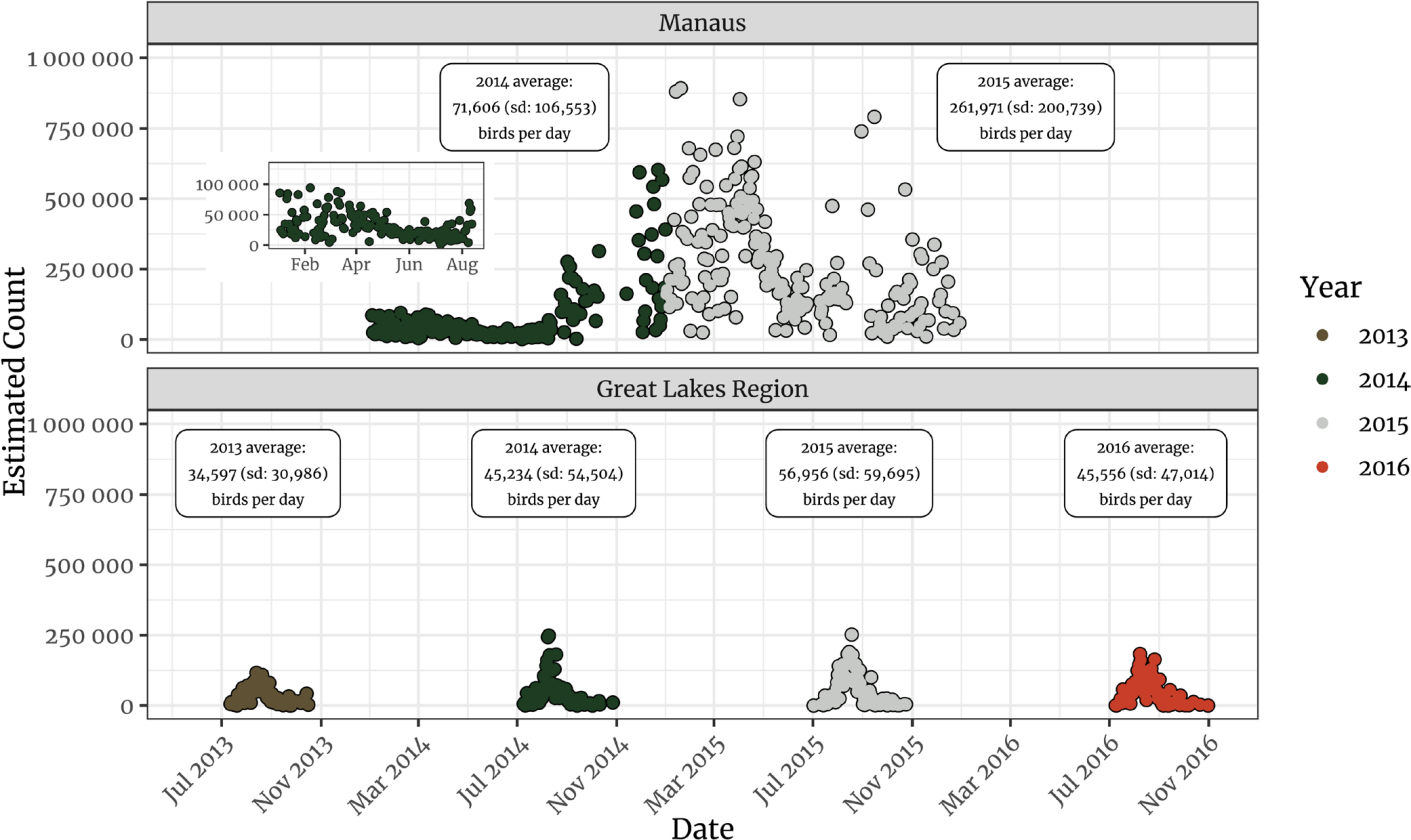
Plots of the daily counts observed in Manaus (upper) and in the Great Lakes region (lower). The area sampled in the Great Lakes region is 8.7 times larger than the area sampled in Manaus. Colors represent different years. Sampling in the Great Lakes occurred from 2013 to 2016, during the period between June 1st and October 31st, whereas in Manaus we examined the entire 2 years of data.

Roosting activity was observed in our study region throughout the year in both 2014 and 2015. In 2014, of the 256 days sampled for the presence/absence of roosts, only 13 were true zeros, which are days when we could observe the entire radar field but still found no evidence of roosts. Similarly, in 2015, of the 276 days sampled for presence/absence of roosts, only seven were true zeros (see Figure 1). The activity pattern in both years, however, was distinct. In 2014, from January to May, when the presence of Purple Martins likely drives roosting activity, we found daily counts ranging from 1487 to 31,649 birds. Activity steadily decreases between May and June and picks up again in July. Starting in August 2014, however, when Purple Martins began arriving in the Amazon, we observed daily counts twice as large as those from the beginning of 2014, with a steady increase until the end of that year. In 2015, the daily counts continued to rise, reaching a peak of 422,538 birds on March 26. The numbers decrease between May and June and start increasing again in late July, similar to what we observed in 2014 (see Figure 1).

**FIGURE 2:**
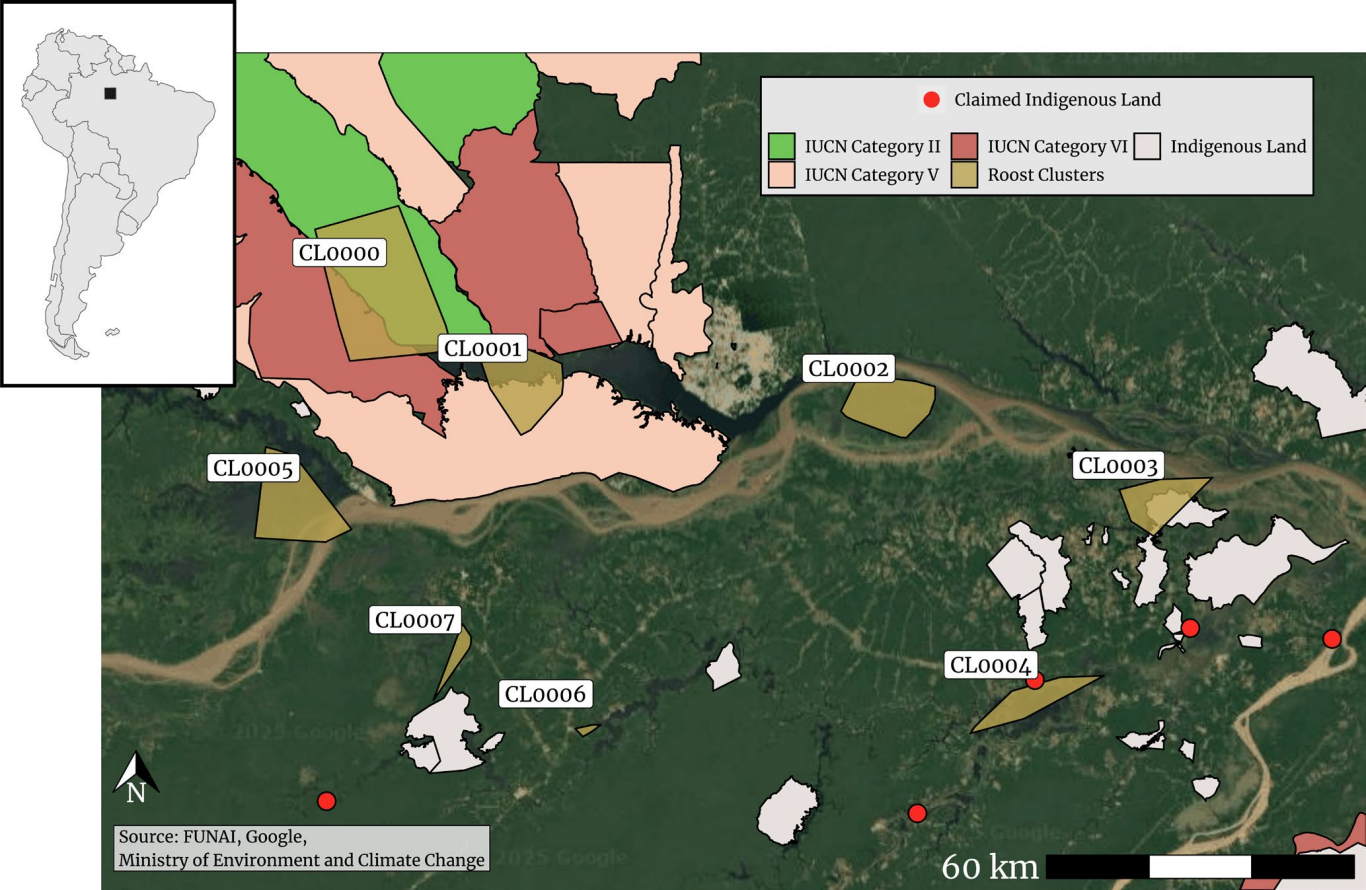
Map of the roost clusters found in Manaus (light gray). Polygons represent either protected land (colored based on IUCN Category) or indigenous land (yellow). Red points represent regions claimed by the indigenous communities of ethnicity Mura since 1997. IUCN Categories: II—national park, III—natural monument or feature, V—protected landscape, and VI—protected area with sustainable use of natural resources.

**FIGURE 3:**
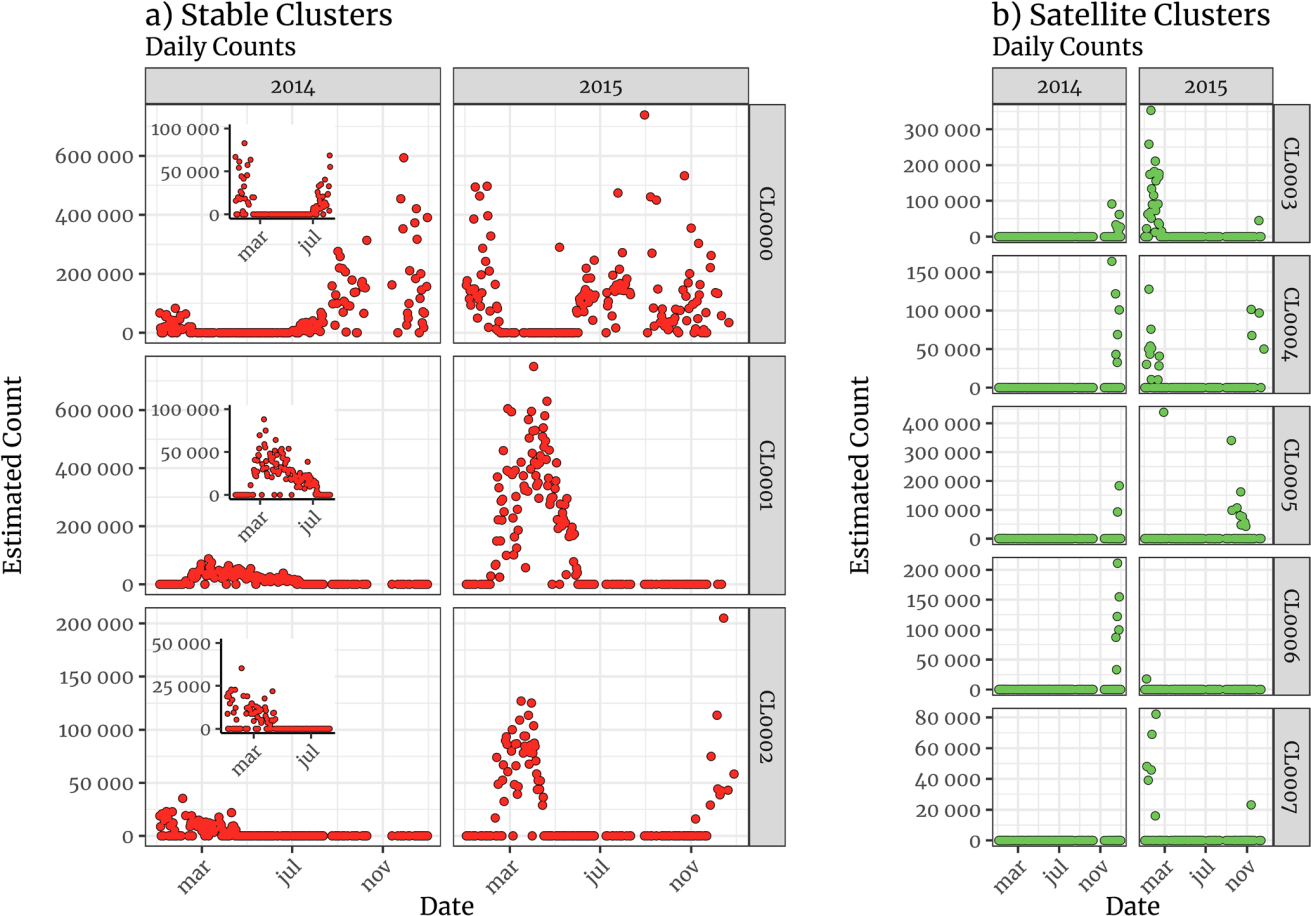
Daily counts of birds in stable (a) and satellite (b) roosts in the region around Manaus. Insets represent counts from January 1st to August 10.

We found eight distinct spatial clusters of roosting activity and, of those, three clusters were systematically used in both years (Figure 2). The remaining clusters, which we will refer to as satellite roosts, were used transiently, especially when we observed the dramatic increase in the daily number of birds in late 2014 and 2015 (see Figure 3). From East to West, the first stable cluster (CL0000) was in the eastern region of the Anavilhanas archipelago, mainly within the Anavilhanas National Park (federally protected land with IUCN Category II). The second stable cluster, CL0001, is the previously documented Negro River roost, described in Arruda (2023) and Oliveira (2021). This roost occurs on Comaru Island, an ephemeral partially inundated island within the limits of a state‐managed protected area reserved for sustainable use (IUCN Category V) between Manaus and Iranduba (see Figure 2). The third stable roost (CL0002) was in the unprotected region surrounding Lago do Rei, in the municipality of Careiro da Várzea (see Figure 2). The largest satellite roost, CL0003, was located on demarcated indigenous land (Terra Indígena Murutinga/Tracajá, see Funai 2024), whereas satellite roost CL0004 is on land claimed by indigenous communities of ethnicity Mura (Ricardo et al. 2023). In both years, we observed that the main roost moved from the Anavilhanas archipelago (CL0000) to Comaru Island (CL0001) just before the peak of the Purple Martin season (February 15, 2014, and February 14, 2015, see Figure 3). It moved back to Anavilhanas on July 1, 2014, much later than it did in 2015 (May 7).

**FIGURE 4:**
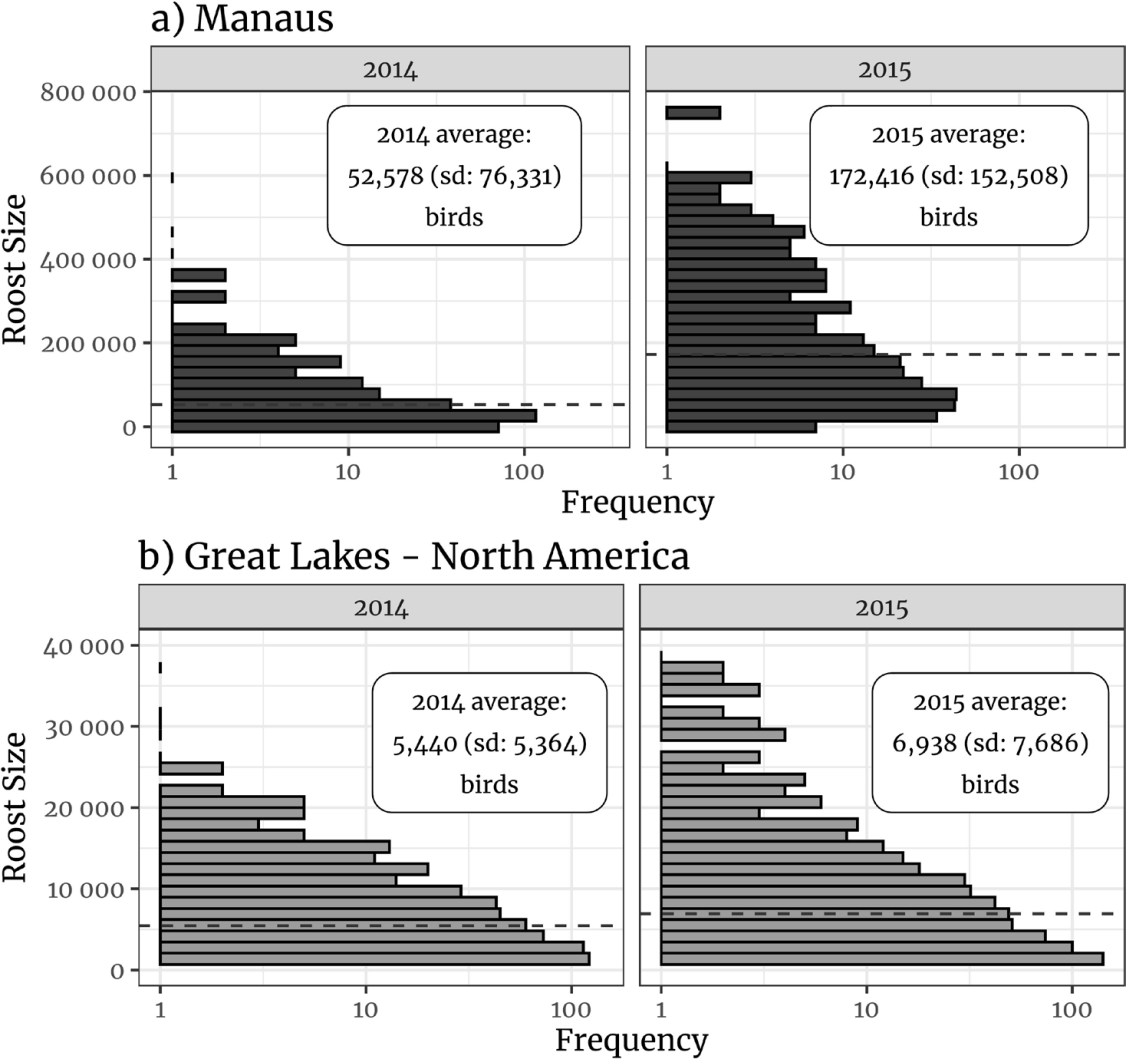
Plots representing the roost size distribution in the Great Lakes (a) and in Manaus (b). The distribution of roost sizes is heavy tailed. The dashed lines represent the means and the *x* axis on both plots is in logarithmic scale.

We subsetted 4 years of data from the Great Lakes (2013–2016), in North America, for our comparison. The area sampled in the Great Lakes corresponds to the union of 12 partially overlapping radar ranges, covering approximately 787,229 ${\mathrm{km}}^{2}$. This area is 8.7 times larger than the area sampled in Manaus (90,000 ${\mathrm{km}}^{2}$). However, we found that the average number of birds per day using the region around Manaus in 2014–71,606 (SD: 106,553) individuals per day—was 1.26 larger than the largest average number of birds per day we found on the Great Lakes region—56,956 (SD: 59,695) individuals per day in 2014 (see Figure 1). Furthermore, in 2015, we observed between 4.62 and 7.57 times more birds per day in the region around Manaus than in the Great Lakes (see Figure 1). At last, with regards to the distribution of roost sizes, we found that Manaus had a much larger average roost size than the Great Lakes region. In 2014, the average roost size in Manaus was approximately 52,578 birds (SD: 76,331) whereas in 2015 that average was 172,416 (SD: 152,508, see Figure 4). In the Great Lakes region, on the other hand, the average roost size was 5440 individuals (SD: 5364) in 2014, and 6938 individuals (SD: 7686) in 2015 (see Figure 4).

## 4 Discussion

We used 2 years of data collected by a weather radar in Manaus to characterize for the first time the phenology of swallow and martin roosting activity in the Amazon Rainforest. We documented the occurrence of eight distinct clusters of roost locations, indicating regions that are repeatedly used by swallows and martins to roost. We found that swallows and martins roost in the region around Manaus throughout the year, with a peak in activity between January and May. This peak corresponds to the annual gathering of Purple Martins in the previously documented Negro River roost, which still occurs on Comaru Island (Arruda 2023; Oliveira 2021). The daily relative abundance pattern observed in both years, however, was distinct: in 2015, our study region hosted substantially more birds than in 2014. Our comparison of nonbreeding roosts in Manaus with pre‐migratory roosts in the Great Lakes region or North America suggests that, on average, roosts gather more birds in Manaus than they do in the Great Lakes, and that the region around Manaus hosted more birds per day than the entire Great Lakes region, which is more than eight times larger. At last, we observed that the roost changed location throughout the year, moving from the Anavilhanas archipelago to Comaru Island and returning to Anavilhanas in May in 2015 and in July in 2014.

Our data confirms previous reports that swallow and martin communal roosts occur in the Amazon throughout the year and are likely gathering different species depending on which migrant is spending their nonbreeding period in the Amazon. In Figure S6, we summarize the number of records per month and per species known to join roosts in the Amazon, based on data available in the Global Biodiversity Network Facility and in the Brazilian community science platform Wikiaves. As we mentioned above, current accounts of roosting activity in Manaus suggest that resident Brown‐chested Martins and Barn Swallows join Purple Martins in the Negro River roost from January to May, though in much smaller numbers (Arruda 2023). Furthermore, historical reports of swallow and martin roosts in the region around Manaus indicate that resident Gray‐breasted Martin may also join these aggregations early in the year (Sick 2001). Since the Amazon receives Austral migrants such as the Southern Martin and the Brown‐chested Martin subspecies *fusca* between July and September, it is possible that these two species are driving roost activity during that period. At last, we note that Purple Martins are seen in low numbers in the Amazon even between June and July (see Figure S6), and on one occasion this species was photographed sharing a roost with Southern Martins in early September (Miranda Júnior and Gomes 2023). Thus, we suggest that communal roosts could act as a link between two migration systems or possibly three, if we find that intertropical migrants such as the Caribbean Martin and the Cuban Martin also join these aggregations when they spend time in the Amazon.

The surge of activity between January and May of 2015 (when compared to the same period in 2014) could either be driven by Purple Martins, or by species that join roosts in smaller numbers, such as resident Brown‐chested Martins subspecies *tapera*, Gray‐breasted Martins, or Barn Swallows (see Figure S6). We consider it unlikely that a sudden increase of that magnitude could be caused exclusively by a resident species. Because of this, we attribute the increase to migrant Purple Martins and, to a lesser degree, to Gray‐breasted Martins, and Barn Swallows. More data are needed to establish if the activity pattern observed in 2015 is still observed today, if it happened just once, or if it happens every few years. We highlight that 2015 observed a record‐breaking El Niño Southern Oscillation event, which is associated with droughts and warmer temperatures in the Amazon (Jiménez‐Muñoz et al. 2016). However, the mechanisms by which El Niño events could influence roosting activity are unclear. These events typically create optimal conditions for the establishment of fires in dryland forests (Holmgren et al. 2001), nevertheless the peak of the wildfire season would be in August and September, after the observed surge of roosting activity. Interestingly, we remark that this specific occurrence of El Niño was marked by temperature anomalies larger than 1°C as early as September of 2014, approximately when our counts start to increase (Jiménez‐Muñoz et al. 2016). Regardless of whether the extreme El Niño event was the cause of the observed pattern, the considerable number of swallows and martins using the Manaus region to roost from January to May in 2015 should be considered when planning human interventions in this area. Nonetheless, our study indicates that the areas used by swallows and martins to roost are well‐protected, except for the region around Lago do Rei and the areas hosting satellite roosts.

The observed difference between roost size and daily number of birds in the Great Lakes region and in the Amazon Rainforest prompts further investigation. Roosting activity in these two regions occurs at different moments of the annual cycle of swallows and martins and thus could be driven by different social and environmental factors. In the Great Lakes region, roosting occurs for a much shorter period, after swallows and martins finish breeding and before they start their southwards migration. In the Amazon, in contrast, roosting activity seems to occur throughout the nonbreeding season, when birds are finishing molting (Niles 1972) and preparing to travel north as soon as possible to compete for better breeding opportunities (Stutchbury et al. 2016). Furthermore, since the Great Lakes region lies in the northern breeding range of most swallows and martins, roosts in that region likely congregate only the portion of the total population that breeds in the northern United States and Canada, whereas, for example, the Amazon is estimated to concentrate approximately 80% of the eastern subspecies of Purple Martins (Fraser et al. 2012). With regard to migration chronologies, we observe that roosting activity in the Great Lakes peaks between July and September. During that same period, we notice activity also starting to increase in the region around Manaus due to the arrival of North American migrants. This confirms findings by Neufeld et al. (2021) that early migrants in the Amazon do not come from the Great Lakes region, since these northern birds have not started their southwards migration yet. Making similar comparisons with more years of data and across a latitudinal gradient in North America could provide insights into where migrant swallows and martins that spend their nonbreeding period in the Amazon breed.

Explaining the change in roost location within a season from the Anavilhanas National Park to Comaru Island requires that we answer two questions: (1) why did the roost move away from Anavilhanas in February, and (2) why does it move back between May and July? Both locations are within the same black water river, the Negro River, where the water level can vary from approximately 3 m in the dry season to 15 m in the wet season (see river level and precipitation plots in the supplements). The area of Comaru Island during the wet season is greatly reduced, which could explain why the birds abandon it, but the difference of almost 2 months between the dates when birds moved up the river in both years can not be explained with current data (see Figure S7a,b for true color renderings of Sentinel data demonstrating the change in surface area of Comaru island). In addition, studies are needed to understand the movement from Anavilhanas to Comaru Island. This movement occurred in mid‐February, 3 months after the river levels started rising (see water level and precipitation plots in the supplements). It is possible that the influx of Purple Martins, paired with roosting substrate reduction due to the rising water levels, could prompt birds to move as a group to the new roosting location, but more data are needed to test this hypothesis.

At last, we remark that recent studies indicate that the intensity and frequency of extreme hydrological events in the Amazon River Basin are increasing, with both droughts and floods becoming more prolonged and intense over time (Correa et al. 2022; Espinoza et al. 2022; Marengo and Espinoza 2015; de Souza et al. 2020; De Faria et al. 2020). These extended periods of extreme conditions disrupt the region's water cycle, which is the main driver of ecological function and diversity in this system. Even though we found that the eight clusters of swallow and martin roost locations are mostly well protected by state and federal regulations, they will still be challenged by these system‐wide threats. Additionally, we note that the population of the city of Manaus grew by approximately 15% between 2010 and 2022 (IBGE 2022, against a 6% national population growth). This urban expansion, if disorganized and unplanned, could bring further disturbance to regional water quality and ecosystem function. Finally, recent studies found that Purple Martins spending their nonbreeding period in Brazil are exposed to potentially harmful levels of mercury from illegal gold mining activities, industrial pollution, and changes in the bioavailability of this metal caused by the construction of hydroelectric dams (Branco et al. 2022; Santos et al. 2025). Within the Amazon Rainforest, there are nine operational S‐band weather radars that could offer us a cost‐effective way to monitor swallow and martin roosting activity—and bird migration more generally—at unprecedented spatial scales. Access to this data could help us understand how migratory birds are responding to these threats and what can be done to mitigate this damage.

## Author Contributions

**Maria C. T. D. Belotti:** conceptualization (lead), data curation (lead), formal analysis (lead), investigation (lead), methodology (lead), project administration (lead), resources (lead), software (lead), validation (lead), visualization (lead), writing – original draft (lead), writing – review and editing (lead). **Victória de Souza Wojahn:** conceptualization (supporting), data curation (supporting), writing – original draft (supporting), writing – review and editing (supporting). **Carlos Augusto Morales Rodriguez:** data curation (equal), methodology (equal), resources (equal), supervision (equal), validation (equal), writing – review and editing (equal). **Kyle G. Horton:** conceptualization (equal), funding acquisition (lead), investigation (equal), methodology (equal), supervision (equal), writing – review and editing (equal).

## Conflicts of Interest

The authors declare no conflicts of interest.

## Supporting information


Data S1.

## Data Availability

The data and scripts used to produce the results of the analyses described in this manuscript can be found in the following link: https://doi.org/10.5281/zenodo.15399430.
