# Supplementary material for: From Spurious Interference to Biological Signal: Repurposing Weather Radars to Monitor and Study the Amazonian Avifauna
Source: Ecol Evol. 2025 Jul 29;15(8):e71860. doi: 10.1002/ece3.71860 (PMC12307130; doi:10.1002/ece3.71860)
Supplement: Supplementary file 1 — Data S1. [file ECE3-15-e71860-s001.docx]

Supplements for: Phenology and spatial distribution of swallow and martin roosts in the Low Negro-Solimões region of the Amazon Rainforest

**Table S1:** Current scientific and common names for the birds mentioned in the paper.

| **Scientific Name** | **English** | **Portuguese** | **Spanish** |
| --- | --- | --- | --- |
| *Chaetura pelagica* | Chimney Swift | andorinhão peregrino | vencejo de chimenea |
| *Oporornis agilis* | Connecticut Warbler | mariquita-de-Connecticut | reinita de Connecticut |
| *Progne subis* | Purple Martin | andorinha-azul | golondrina purpúrea |
| *Progne tapera* | Brown-chested Martin | andorinha-do-campo | golondrina parda |
| *Progne elegans* | Southern Martin | andorinha-do-sul | golondrina sureña |
| *Progne chalybea* | Grey-breasted Martin | andorinha-grande | golondrina pechigrís |
| *Progne dominicensis* | Caribbean Martin | andorinha-do-Caribe | golondrina caribeña |
| *Progne cryptoleuca* | Cuban Martin | andorinha-cubana | golondrina cubana |
| *Hirundo rustica* | Barn Swallow | andorinha-de-bando | golondrina común |
| *Tachycineta bicolor* | Tree Swallow | andorinha-das-árvores | golondrina bicolor |


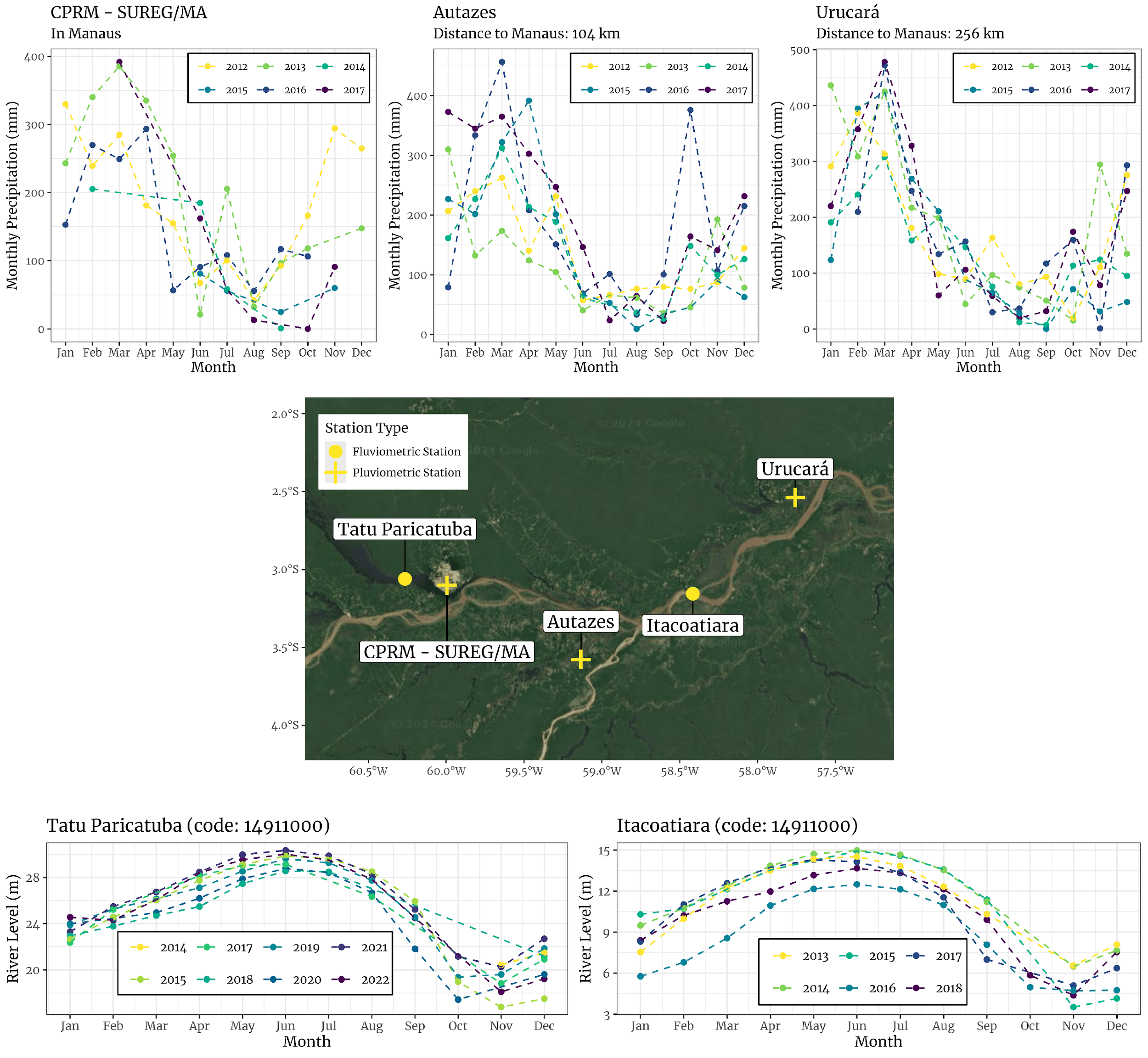


**Figure S1:** This panel characterizes the so-called monomodal flood pulse that regulates biogeochemical cycles in the Amazon basin. The upper plots contain historical measurements of monthly precipitation (measured in millimeters) from three pluviometric stations up to 256 km from Manaus. The bottom plots represent historic water level measurements collected by monitoring instruments close to our study area. The map depicts instrument locations. The data is collected, processed, and maintained by the Agência Nacional de Águas of the Brazilian government.


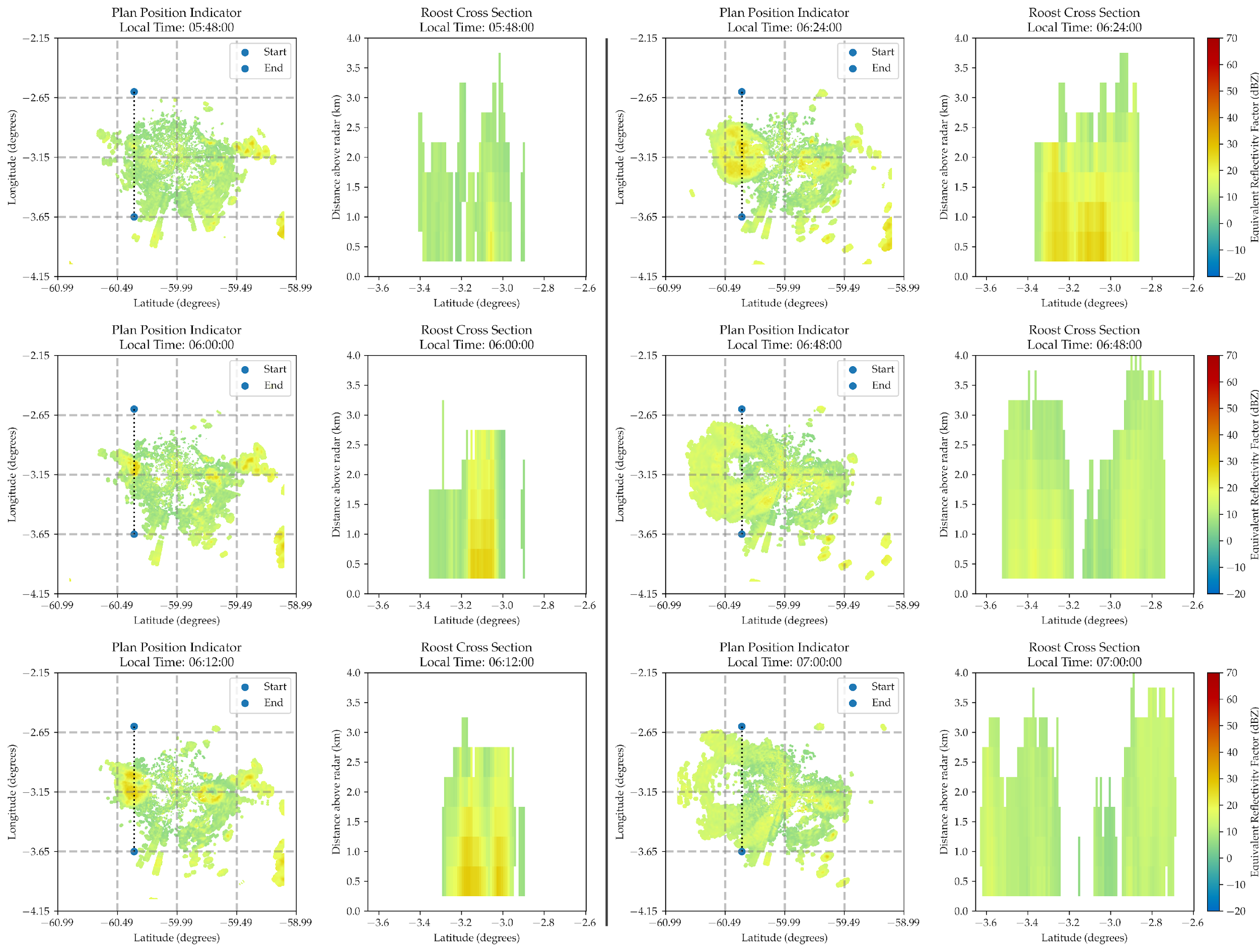


**Figure S2:** This panel shows subsequent scans made on March 3, 2015. For each pair of plots, figures on the left are plan position indicators with the radar at the center, while figures on the right are cross sections of the main roost dispersing from Comaru Island. The cross section was made at the line represented on the plan position indicator.


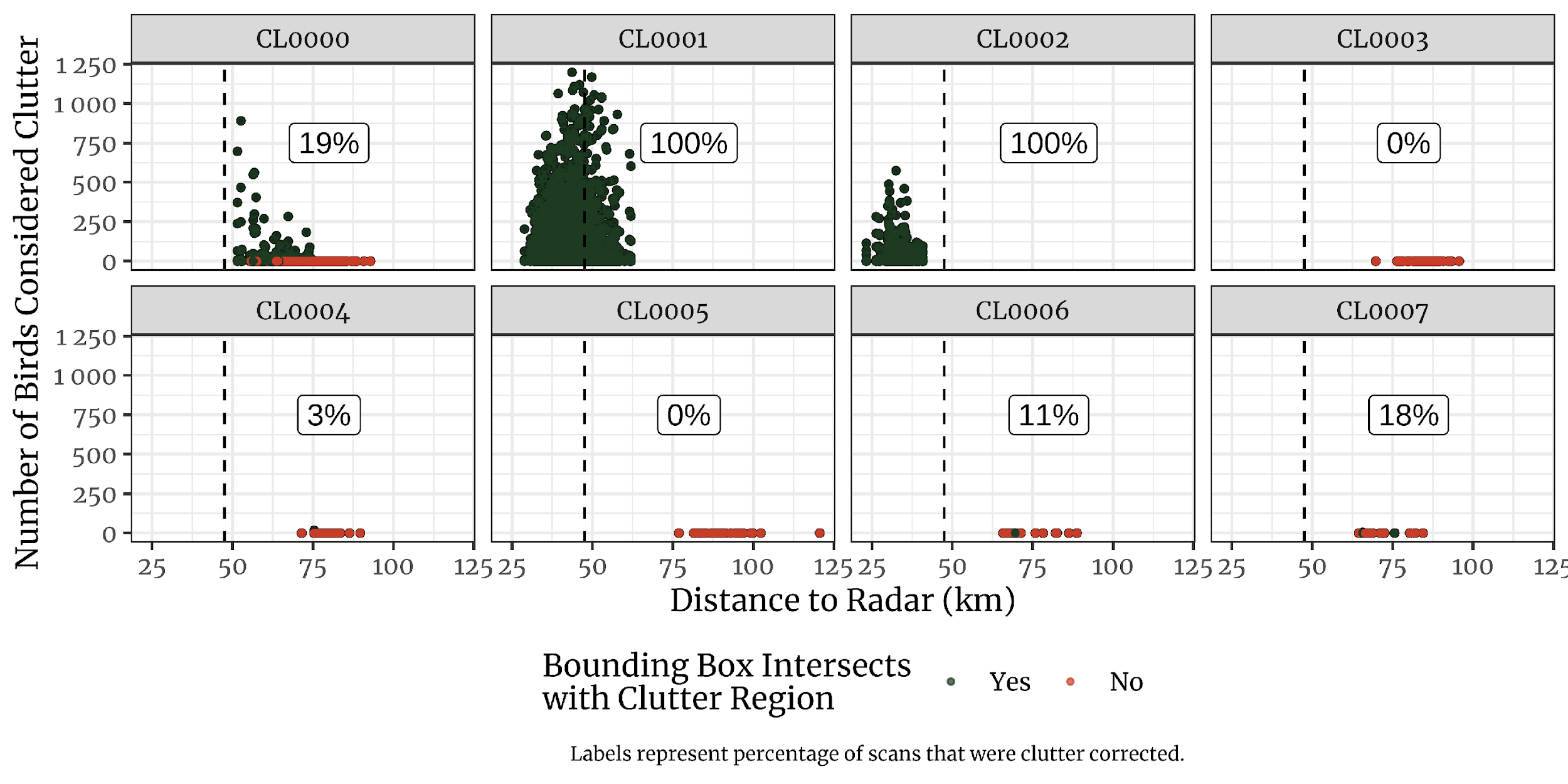


**Figure S3:** The plots represent, for each cluster of roosts, the number of birds that were removed by our clutter filtering procedure as a function of distance between the radar and the centroid of the bounding box (measured in kilometers). The labels contain percentages of the total number of sweeps that were effectively filtered. The points are colored in dark green if the bounding box intersects the clutter mask, and in red if they do not. Notice that even though the centroid of a bounding box might be outside of the mask range of 27.5 km, the bounding box itself can still intersect the clutter mask.


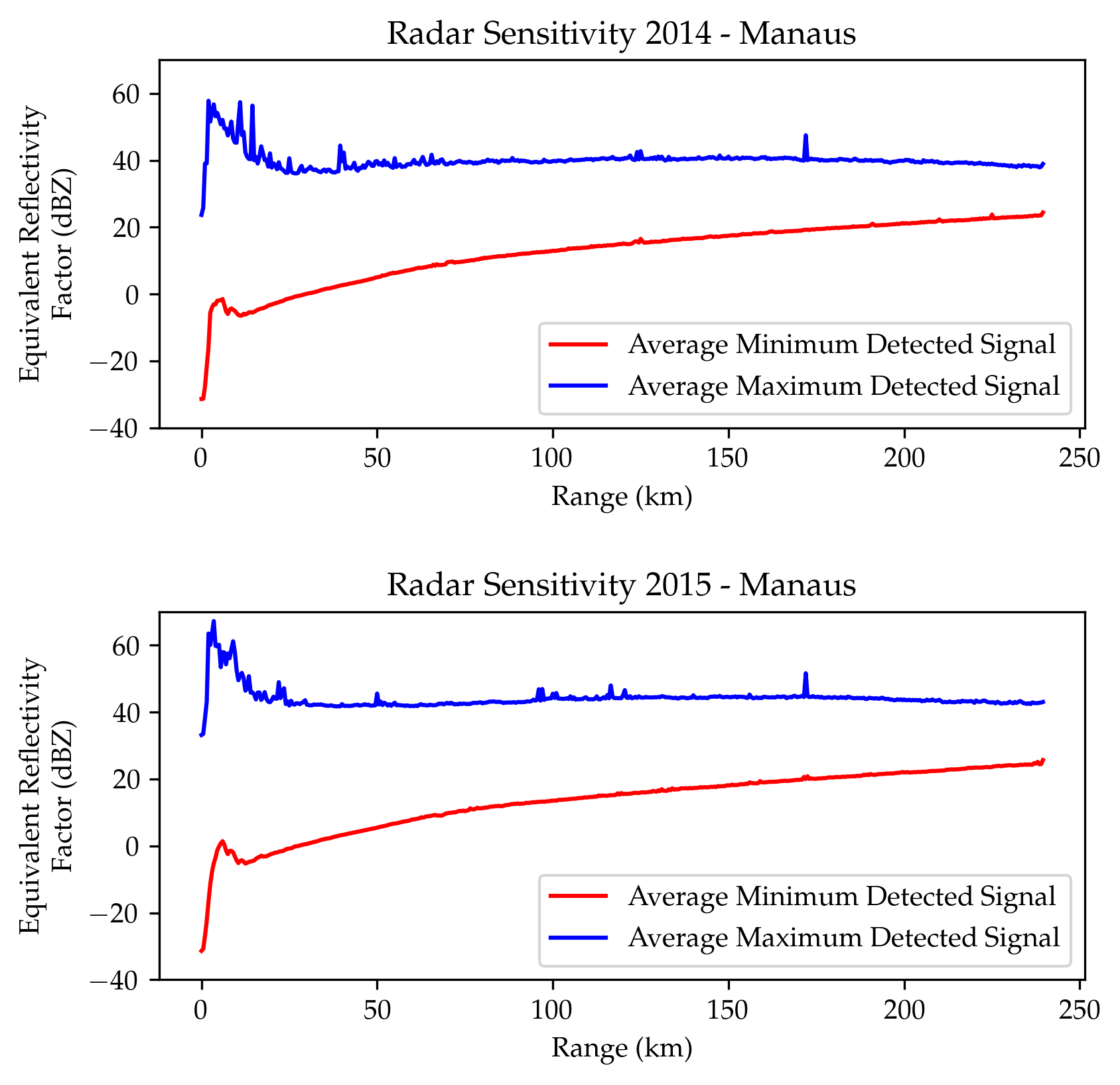


**Figure S4:** Plots representing the minimum (red) and maximum (blue) detected equivalent reflectivity factor in dBZ for each range gate, averaged across all scans made by the Manaus radar within our study period for each year. We note that the curves for both years are similar, indicating that there were no instrument changes between 2014 and 2015.


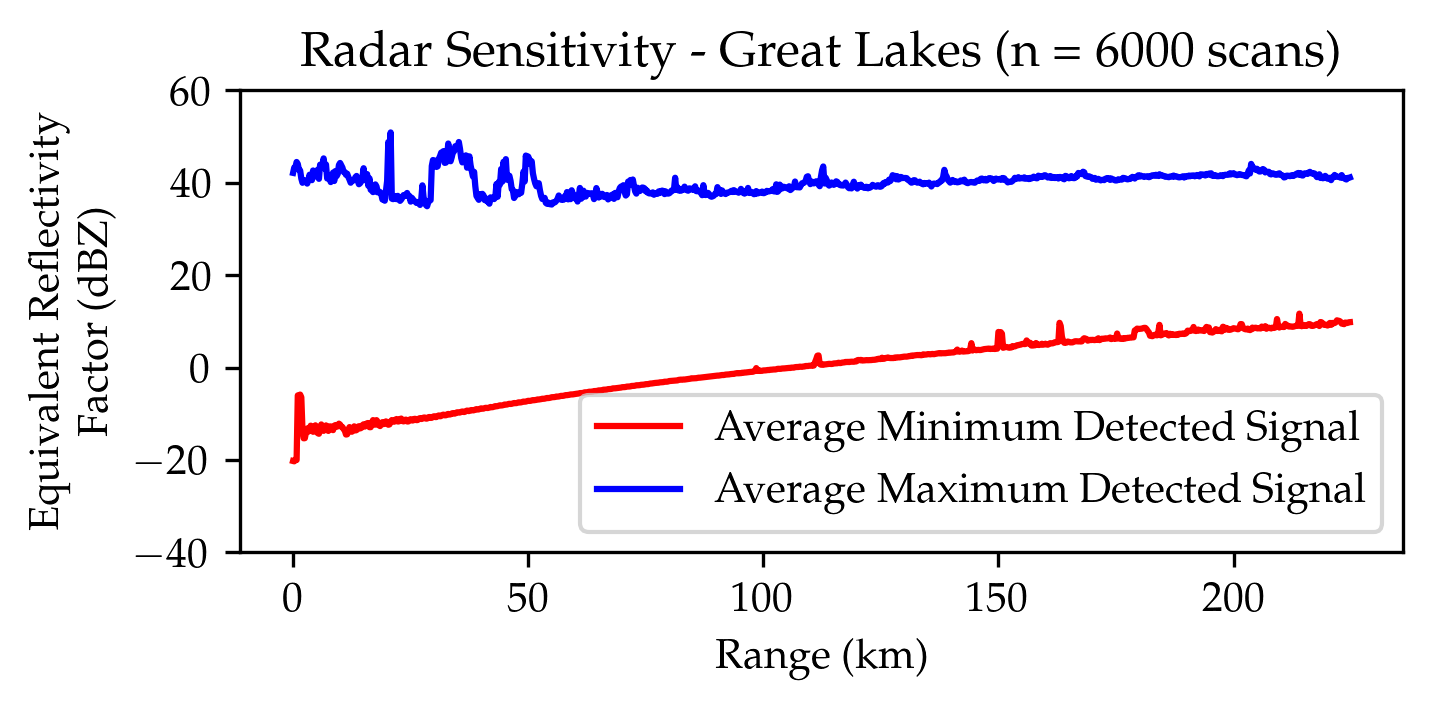


**Figure S5:** Plos representing the minimum (red) and maximum (blue) detected equivalent reflectivity factor in dBZ for each range gate, averaged across a sample of 6000 scans made at dawn by twelve radar stations in the Great Lakes region from 2001 to 2022.


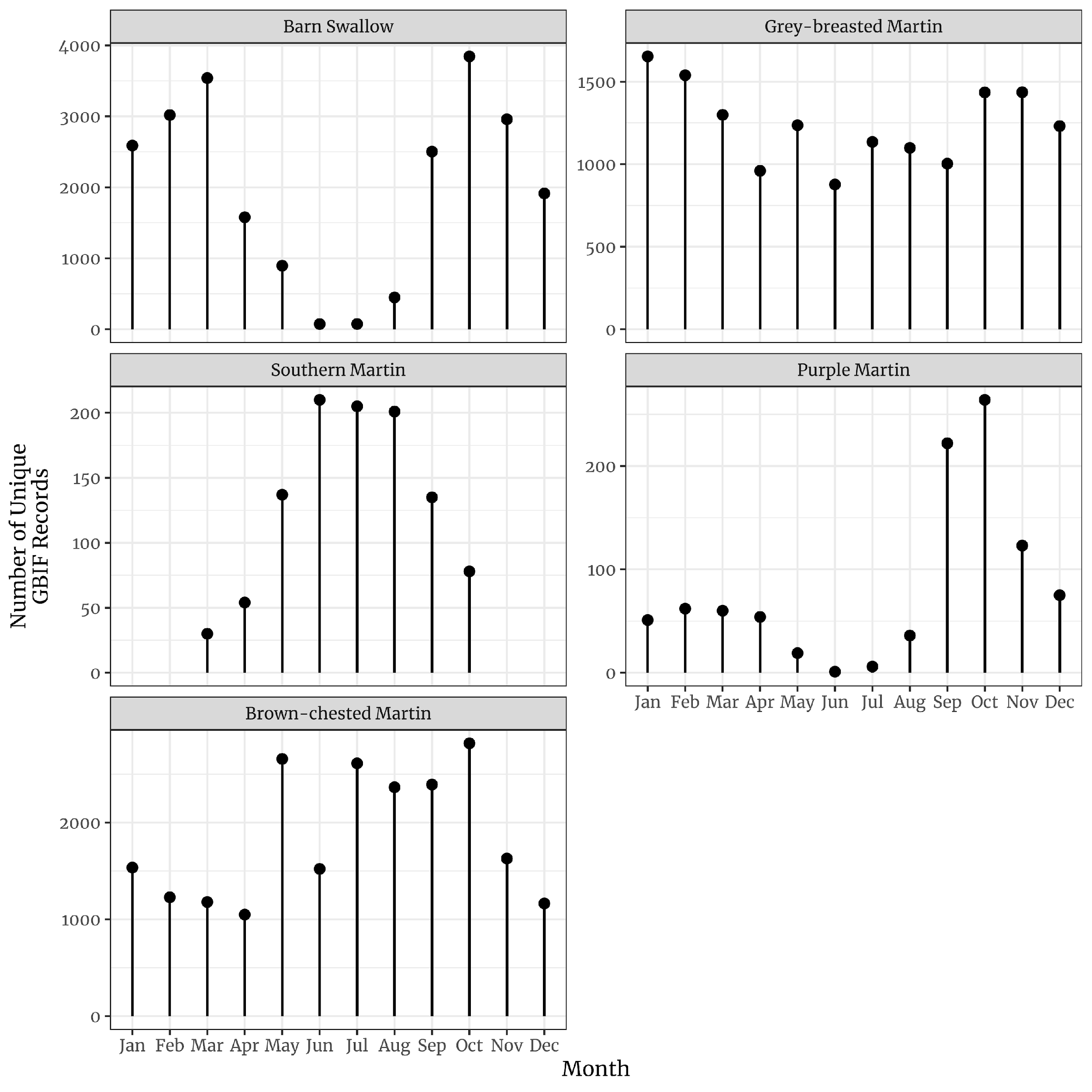


**Figure S6:** Plots representing the number of records available in the Global Biodiversity Information Facility and in the Brazilian community science platform called Wikiaves for each of the species known to join roosts in larger numbers in the Amazon Rainforest. Purple Martins are Nearctic-Neotropical migrants (Gbif 2024d). Southern Martins and Brown-chested Martins subspecies fusca are Austral migrants (Gbif 2024e, 2024c). Gray-breasted Martins and Barn Swallows are partial migrants, and Brown-chested Martins subspecies tapera are residents in the Amazon (Gbif 2024a, 2024b). Data is collected from museums and community science databases such as iNaturalist and eBird, and represent occurrence-only observations from 1819 to 2024. Individual records can represent hundreds of thousands of individuals.

1. Comaru Island (highlighted) in the Negro River.
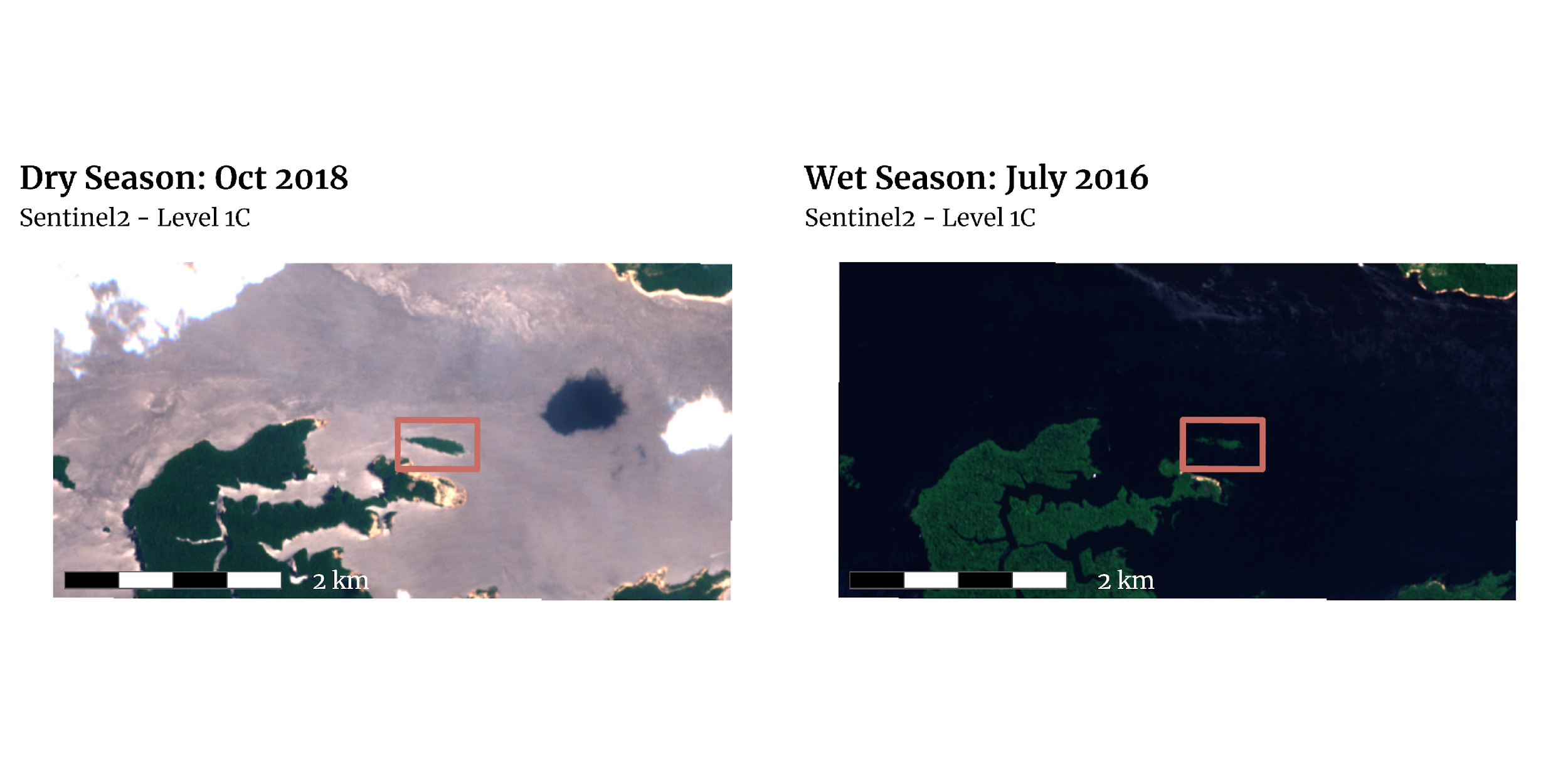

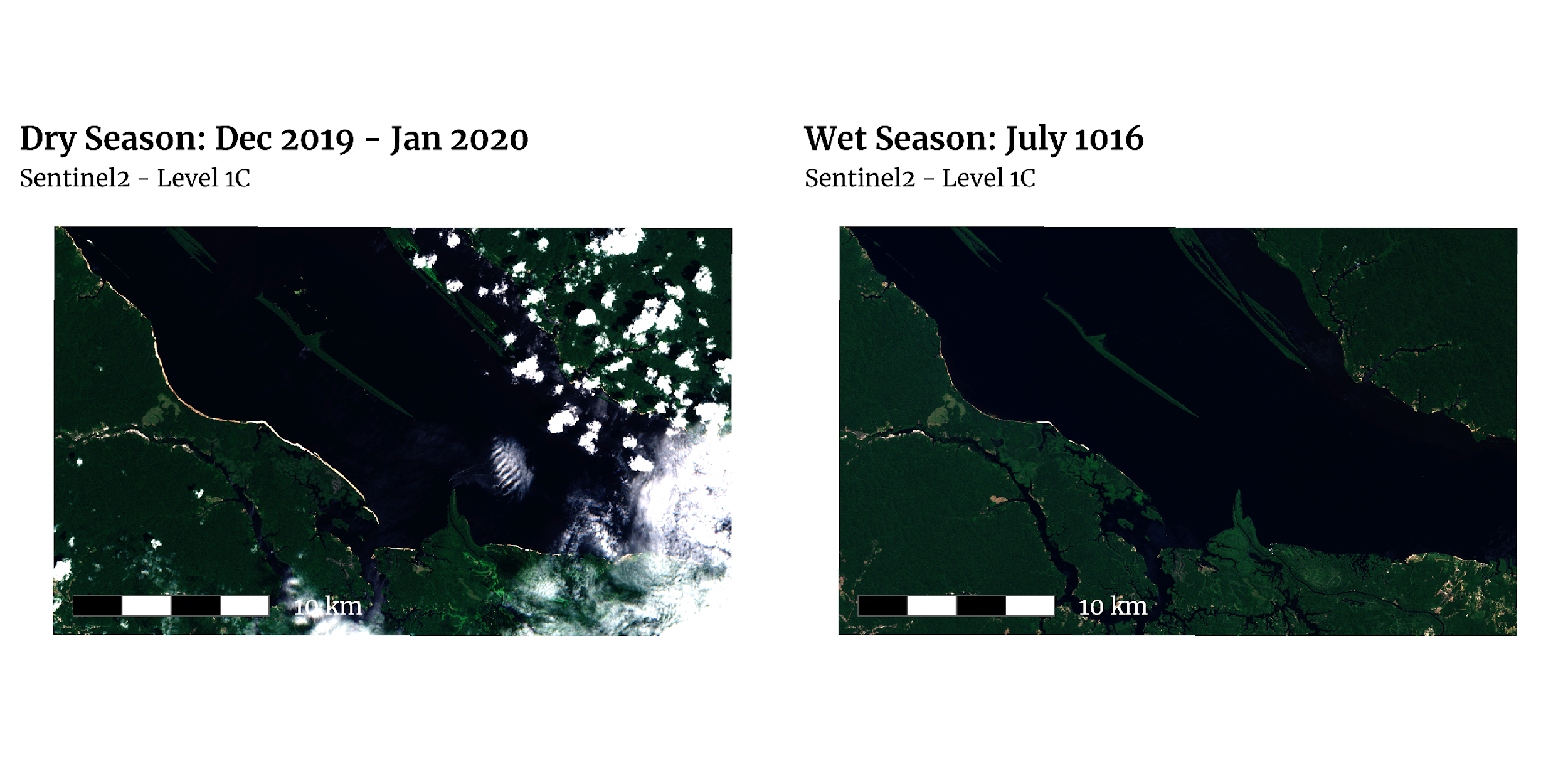

2. Anavilhanas National Park in the Negro River

**Figure S7:** True color renderings of Sentinel reflectance data (10 m resolution) (European Space Agency 2022). We created mosaics from month/year combinations with minimal cloud blockage. The peak of the dry season is typically between October and November, whereas the peak of the wet season is between June and July.

# References

GBIF. 2024a. “Hirundo Rustica - Occurrence Download.” The Global Biodiversity Information Facility. <https://doi.org/10.15468/dl.syk7gh>.

———. 2024b. “Progne Chalybea - Occurrence Download.” The Global Biodiversity Information Facility. <https://www.gbif.org/occurrence/download/0025138-240626123714530>.

———. 2024c. “Progne Elegans - Occurrence Download.” The Global Biodiversity Information Facility. <https://doi.org/10.15468/dl.qgrem4>.

———. 2024d. “Progne Subis - Occurrence Download.” The Global Biodiversity Information Facility. <https://doi.org/10.15468/dl.j683da>.

———. 2024e. “Progne Tapera - Occurrence Download.” The Global Biodiversity Information Facility. <https://doi.org/10.15468/dl.pjvagv>.

European Space Agency, ESA. 2022. “Sentinel-2 MSI Level-1C TOA Reflectance.” European Space Agency. <http://dx.doi.org/10.5270/S2_-742ikth>.
